# Natural deep eutectic solvents: cytotoxic profile

**DOI:** 10.1186/s40064-016-2575-9

**Published:** 2016-06-29

**Authors:** Maan Hayyan, Yves Paul Mbous, Chung Yeng Looi, Won Fen Wong, Adeeb Hayyan, Zulhaziman Salleh, Ozair Mohd-Ali

**Affiliations:** University of Malaya Centre for Ionic Liquids (UMCiL), University of Malaya, 50603 Kuala Lumpur, Malaysia; Department of Civil Engineering, University of Malaya, 50603 Kuala Lumpur, Malaysia; Department of Chemical Engineering, University of Malaya, 50603 Kuala Lumpur, Malaysia; Department of Medical Microbiology, University of Malaya, 50603 Kuala Lumpur, Malaysia; Department of Pharmacology, University of Malaya, 50603 Kuala Lumpur, Malaysia; Institute of Halal Research University of Malaya (IHRUM), Academy of Islamic Studies, University of Malaya, 50603 Kuala Lumpur, Malaysia; UiTM Medical Specialist Centre, University of Technology MARA, Jalan Hospital, 47000 Sungai Buloh, Selangor, Malaysia

**Keywords:** Natural deep eutectic solvents, Green solvents, Ionic liquids, Choline chloride, Cytotoxicity, Cancer cell line, COSMO-RS

## Abstract

The purpose of this study was to investigate the cytotoxic profiles of different ternary natural deep eutectic solvents (NADESs) containing water. For this purpose, five different NADESs were prepared using choline chloride as a salt, alongside five hydrogen bond donors (HBD) namely glucose, fructose, sucrose, glycerol, and malonic acid. Water was added as a tertiary component during the eutectics preparation, except for the malonic acid-based mixture. Coincidentally, the latter was found to be more toxic than any of the water-based NADESs. A trend was observed between the cellular requirements of cancer cells, the viscosity of the NADESs, and their cytotoxicity. This study also highlights the first time application of the conductor-like screening model for real solvent (COSMO-RS) software for the analysis of the cytotoxic mechanism of NADESs. COSMO-RS simulation of the interactions between NADESs and cellular membranes’ phospholipids suggested that NADESs strongly interacted with cell surfaces and that their accumulation and aggregation possibly defined their cytotoxicity. This reinforced the idea that careful selection of NADESs components is necessary, as it becomes evident that organic acids as HBD highly contribute to the increasing toxicity of these neoteric mixtures. Nevertheless, NADESs in general seem to possess relatively less acute toxicity profiles than their DESs parents. This opens the door for future large scale utilization of these mixtures.

## Background

The use of volatile organic compounds (VOCs) has left in its wake countless considerations, most of which associated with safety and toxicity issues (Bushnell et al. 2007). Therefore, for various chemical and biological industries, one of the most pressing concern remains the development of ‘greener’, lower cost, and more efficient solvents. The discovery of ionic liquids (ILs) seemed to provide the solution to this predicament. ILs being low-melting organic salts composed of ionic species, which are often found in liquid state at temperatures below 100 °C (Ru and Konig 2012). ILs are characterized by a number of attractive properties such as high thermal stability, non-flammability, high solvability, chemical stability, low volatility and tunability (Sowmiah et al. 2009). Notwithstanding their impressive contributions in various processes such as biotransformations (Domínguez de María and Maugeri 2011), biodiesel production (Ullah et al. 2015), extraction processes (Pereira et al. 2015), active pharmaceutical ingredients (Ferraz et al. 2011), and biomass treatment (da Costa Lopes et al. 2013); the use of ILs has often been marred with issues pertaining to high cost of synthesis, purification requirements, and toxicity (Gorke et al. 2010). The ambiguity surrounding their use has led to the emergence of an alternative class of solvents called deep eutectic solvents (DESs). As eutectics, DESs exhibit freezing points lower than those of their chief components (salt and HBD) (Smith et al. 2014). This depression in temperature is the result of the charge delocalization occurring via hydrogen bonding between the anion of the salt and the HBD (Ru and Konig 2012). DESs have been introduced as viable replacements of ILs because, on top of possessing similar physicochemical aspects, they offer several other advantages, such as the low cost of their starting materials, ease of preparation, and no waste generation (Tang and Row 2013). As a result, they have been used in a wide number of applications such as extraction processes (Qi et al. 2015), biotransformations (Wu et al. 2014), nanoparticles assembly (Wagle et al. 2014), preservation of biomolecules (Dai et al. 2015), upstream and downstream biodiesel processing (Hayyan et al. 2010, 2013a, 2014), electrodeposition (Ru et al. 2015), and organic synthesis (Zhang et al. 2012).

The most encountered DESs are based on ChCl, which revolves around choline. Choline is a known component of Vitamin B, and plays important metabolic functions (Florindo et al. 2014). The qualifications of choline as a safe ingredient led to the assumption that DESs possess negligible toxicity/cytotoxicity profiles.

However, the leading cytotoxic assessments of DESs showed that they can be lethal to both terrestrial and marine organisms (bacteria and brine shrimp) (Hayyan et al. 2013b, c). Ammonium-based DESs, namely: [ChCl]-[Glycerol], [ChCl]-[Ethylene glycol], [ChCl]-[Triethylene glycol], [ChCl]-[Urea]; and methyltriphenyl phosphonium bromide (MTPB)-based DESs such [MTPB]-[Glycerol], [MTPB]-[Ethylene glycol], and [MTPB]-[Triethylene glycol], were used during these investigations. Both ammonium and phosphonium-based DESs were found toxic to brine shrimp, but only phosphonium-based DESs exhibited bacterial toxicity. The cytotoxicity of both ammonium and phosphonium-based DESs was higher than those of their individual components (Hayyan et al. 2013b, c). Therefore, the authors concluded that although ChCl and MTPB salts are not devoid of toxicity; their association with a HBD during DESs’ preparation increases the eutectics’ cytotoxicity considerably. This conclusion has been recurrent throughout several cytotoxic assessments of DESs (Hayyan et al. 2015; Radošević et al. 2015; Wen et al. 2015).

The HBD has proven to be of significant importance with regards to DESs’ cytotoxic profiles. Recently, Radošević et al. (2015) examined the cytotoxic profile of [ChCl]-[Glucose], [ChCl]-[Glycerol], and [ChCl]-[Oxalic acid] on channel catfish ovary (CCO) fish cell line and the human breast adenocarcinoma cell line (MCF-7). Their results showed that the [ChCl]-[Oxalic acid] DES exhibited a significantly higher toxicity (EC_50_ < 5 mM) compared to the remaining ChCl-based DESs (EC_50_ > 10 mM). These results reinforced the importance of a careful selection of the HBD prior DESs synthesis. In yet another study, among four ChCl-based DESs namely, [ChCl]-[Urea], [ChCl]-[Acetamide], [ChCl]-[Ethylene glycol], and [ChCl]-[Glycerol]; the [ChCl]-[Ethylene glycol] DES was shown to have the highest toxicity (Wen et al. 2015).

The cytotoxic mechanism of DESs outlines an increased membrane porosity due to the continuous DESs’ induced damage of the plasma membrane (Hayyan et al. 2015). Accordingly, upon penetration, DESs’ species effectively contribute to the increase of reactive oxygen species (ROS) concentrations, hereby subjecting the cell to increasing oxidative stress. The end-point of this cascade involves the complete destruction of the cell through apoptosis (Hayyan et al. 2015).

The key to producing less toxic DESs may reside in the use of materials of natural origin. Recently, Choi et al. (2011) revealed that a number of primary metabolic substances (e.g. sugars, amino acids, choline, and some organic acids) form intracellular eutectic mixtures to assist plants during specific developmental stages (germination, cryopreservation). These eutectics provide cells with a third type of solvent/media, completely different from lipids and water. The presence of these eutectic mixtures—termed natural deep eutectic mixtures (NADESs)—intracellularly, entails their cellular tolerance and presume safer cytotoxic profiles. Recent studies have provided a list of the composition of these mixtures as well as their molar ratios (Choi et al. 2011; Dai et al. 2013).

One of the fundamental precepts of this class dictates that if cellular media produce NADESs, the propensity for cytotoxicity must be minimal. Paiva et al. (2014) briefly investigated the cytotoxic profile of several NADESs namely, [ChCl]-[Glucose], [ChCl]-[Citric acid], [ChCl]-[sucrose], [ChCl]-[Tartaric acid], [ChCl]-[Xylose], [Citric acid]-[Glucose], [Citric acid]-[Sucrose], [Glucose]-[Tartaric acid] at various molar ratios. Using fibroblast-like cells, the authors assessed cellular viability following NADESs’ treatment. The results pointed once again to the role of the HBD (organic acids) as major enhancer of cytotoxicity, because the most toxic NADESs were [Glucose]-[Tartaric acid], [ChCl]-[Tartaric acid], [ChCl]-[Citric acid], and [Citric acid]-[Glucose]. Understanding the various interactions or forces resulting from the association of NADESs chief elements can provide further elucidation of their resulting cytotoxic profiles.

A recent study has shown that NADESs physical properties can be tailored by adding water as a tertiary component. The authors showed that the strong hydrogen interactions within NADESs (accounting for their high viscosities) could be reduced upon addition of water (<50 % v/v). In fact, the resulting viscosities were found to be as low as those of water and other common VOCs (Dai et al. 2015). Consequently, water-based NADESs may represent yet another alternative to DESs of high viscosities, poor conductivities and higher toxicities.

Hence, one of the objectives of this study was to investigate the effect of water on the cytotoxic profiles of NADESs by exploring the impact ternary NADES systems (containing water) have on several cancer cell lines. The understudied eutectics were ChCl-based NADESs prepared using five HBDs, namely, fructose, glucose, sucrose, glycerol, and malonic acid. Alternatively, we aimed to compare the effect of biomaterials against that of organic acids on mammalian cells. Lastly, we sought to model the interactions between NADESs and cellular membranes using COSMO-RS computational methodology in order to understand NADESs cytotoxic mechanism.

## Methods

### Chemicals and materials

**Fig. 1:**
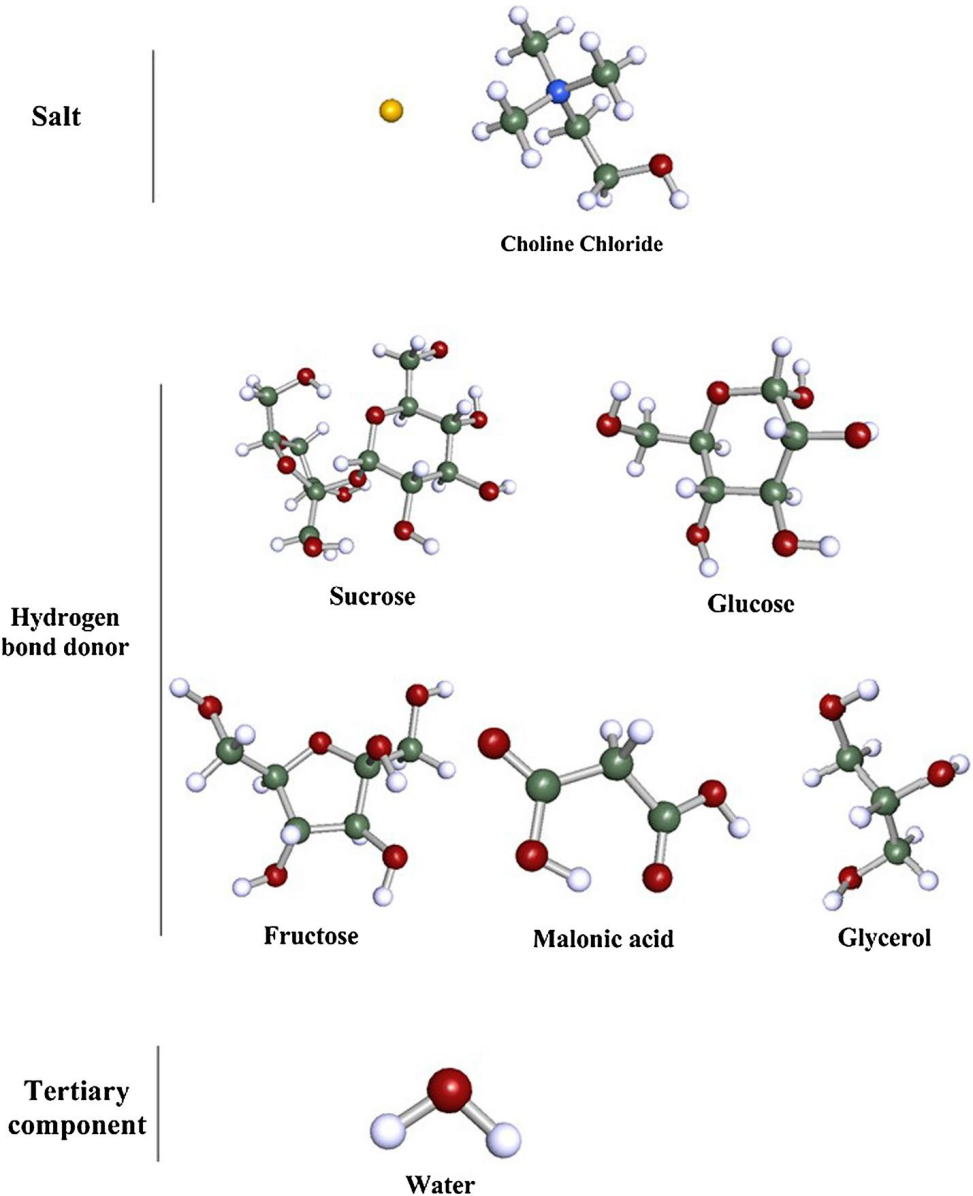
Structure of the individual components of the NADESs used in this study

The chemicals used for NADESs preparation were purchased from both Merck (sucrose, fructose and glucose) and Sigma-Aldrich (ChCl and malonic acid), Fig. 1. The purity of these chemicals was higher than 99 %.

The human cervical cancer cell line (HelaS3), the human ovarian cancer cell line (CaOV3), and the mouse skin cancer cell line (B16F10) were purchased from the American Type Culture Collection (ATCC, Manassas, VA). The human breast cancer cell line (MCF-7) was obtained from Cell Lines Service (300273; Eppelheim, Germany).

Both the Dulbecco’s Modified Eagle Medium (DMEM) and the Roswell Park Memorial Institute medium (RPMI 1640) were obtained from Life Technologies, Inc., Rockville, MD. Fetal bovine serum (FBS) was supplied by Sigma-Aldrich.

### Preparation of NADESs

Table 1 illustrates the composition, molar ratios and symbols of the NADES used throughout this study. The preparation method is similar to those previously described in the literature (Hayyan et al. 2013c).

**Table 1:** Compositions, symbols, and molar ratios of the NADESs used in this study

Salt	HBD	Add-on	Molar ratio	Appearance	Symbol
ChCl	Fructose	Water	5:2:5	Moderately viscous liquid	NADES_1_
ChCl	Glucose	Water	5:2:5	Moderately viscous liquid	NADES_2_
ChCl	Sucrose	Water	4:1:4	Moderately viscous liquid	NADES_3_
ChCl	Glycerol	Water	1:2:1	Lightly viscous liquid	NADES_4_
ChCl	Malonic acid	–	1:1	Moderately viscous liquid	NADES_5_

### Cell culture

HelaS3, CaOV3, and B16F10 were grown in DMEM supplemented with 10 % FBS, 1 % penicillin and streptomycin. MCF-7 were grown in RPMI supplemented with 10 % FBS, 1 % penicillin and streptomycin. The cells were kept in culture flasks inside an incubator providing a humidified atmosphere of 37 °C, with 5 % CO_2_. The cells were grown to a necessary confluence of 70–80 %, necessary for the 3-(4, 5-dimethylthiazolyl-2)-2, 5-diphenyltetrazolium bromide (MTT) viability assay.

### MTT viability assay

The MTT cell viability assay was performed as previously described (Hayyan et al. 2015). The IC_50_ values were obtained from an average of at least 3 independent experiments. The standard error of the mean (SEM) derived from the repeated experiments were used to derive the variations from the average IC_50_ values. The statistical analysis was performed using Graph Pad Prism 5 software. Statistical significance was defined when P < 0.05.

### Computational methodology for COSMO-RS

#### Molecular geometry optimization

The geometry optimization of all species involved in this study was performed using the Turbomole program package. Using this program, the basic structure of the target molecule was drawn first. After which, geometry optimization was performed at the Hartree–Fock level and 6-31G* basis set. The generation of cosmo file was then conducted through a single-point calculation by using DFT with Becke–Perdew and the Triple-ζ Zeta Valence Potential (TZVP) basis set. Finally, the *cosmo files* were exported to the COSMOthermX program with parameterization BP_TZVP_C30_1301.ctd.

#### DES representation in COSMOtherm-X

Since a single DES is composed of more than one molecule, employing its representation method in the COSMOtherm-X program is crucial. In this study, the electroneutral approach was adopted, whereby the DES was represented in COSMO-RS according to the mole composition of their constituents shown in Table 1 [the salt cation, salt anion, and hydrogen bond donor (HBD)]. Membrane phospholipids were designed according to the same principle; that is using the most basic composition of their constituents (Table 2).

**Table 2:** Composition of cellular membrane and ratio (Harwood and Weselake 2015)

Cell membrane elements	Composition and ratio			
	Fatty acid	Alcohol	Metabolites	Functional group
Phosphatidylcholine	Palmitic acid (2)	Glycerol (1)	Choline (1) Phosphate (1)	Phosphate (1)
Phosphatidylethanolamine	Linoleic acid (1); palmitic acid (1)	Glycerol (1)	Ethanolamine (1) Phosphate (1)	Phosphate (1)
Phosphatidylserine	Stearic acid (1); cervonic acid (1)	Glycerol (1)	Serine (1) Phosphate (1)	Phosphate (1)
Sphingomyelin	Oleic acid (1)	–	Choline (1) Phosphate (1)	Phosphate (1)
Glycolipids	Oleic acid (1)	–	Glucose (1) Sphingosine (1)	Phosphate (1)

## Results and discussion

The cytotoxicity of the five understudied NADESs was assessed on various human and mice cancer cell lines, namely, HelaS3, CaOV3, MCF-7, and B16F10. Table 3 illustrates the IC_50_ values obtained. The results indicate the following decreasing order of toxicity for HelaS3, MCF-7, and B16F10 cell lines: NADES_5_ > NADES_3_ > NADES_1_ > NADES_2_ > NADES_4_. In CaOV3 case, NADES_2_ was more toxic than NADES_1_ resulting in a slightly different trend: NADES_5_ > NADES_3_ > NADES_2_ > NADES_1_ > NADES_4_. However, if the SEM of the IC_50_ values are included, the resulting IC_50_ intervals of both NADES_1_ (198.5–213.5 mM) and NADES_2_ (185.5–200.5 mM) overlap, as the end values are close to one another.

**Table 3:** IC_50_ of the studied NADESs on various cell lines

Solvent	IC_50_ (mM)			
	Hela S3	CaOV3	MCF-7	B16F10
NADES_1_	177 ± 7.3	206 ± 7.5	127 ± 9.22	195 ± 7.7
NADES_2_	182 ± 7.6	193 ± 7.5	186 ± 7.9	211 ± 8
NADES_3_	166 ± 5.8	154 ± 5.6	150 ± 5.5	136 ± 5.7
NADES_4_	427 ± 11	483 ± 11	457 ± 11	340 ± 10.3
NADES_5_	20 ± 8.4	15 ± 8.2	35 ± 8.3	35 ± 8.8

Overall, we noticed a trend between NADESs’ cytotoxicity and various factors namely the cellular requirements of cancer cells, the physical properties of NADESs (especially viscosity); the addition of water; and the nature of NADESs’ raw materials as well as their interactions with the different functional groups present on the cell surface.

The merits of most DESs stem from the qualifications of ChCl; with specific referral to the metabolism and function of choline in mammalian cells. Choline is the preferred cellular raw material used for the synthesis of cellular membranes phospholipids, namely phosphatidylcholine, and sphingomyelin (Lodish et al. 2000; Plagemaen 1971). Consequently, ChCl has been classified as a salt of relatively safe profile (although high intake is associated with adverse conditions). However, the DESs cytotoxic profiles obtained thus far do not share the negligible cytotoxic labeling of ChCl. This has prompted the examination of the role of the HBD in these profiles.

From a cellular perspective, fructose, glucose, sucrose (50 % glucose and 50 % fructose), and glycerol are essential carbohydrates whose metabolism provide energy required for various cellular functions. Upon adsorption, fructose and glucose undergo glycolysis if energy is needed, or are stored as glycogen. The glycolytic pathway for these molecules leads to either the pentose phosphate pathway (for nucleic acid synthesis), the mitochondrial tricarboxylic acid pathway (for energy production), or de novo lipogenesis (for fatty acids synthesis). Cancer cells, especially, require more energy than normal cells, given their abnormal and exponential growth features. Therefore, they use a higher amount of energy or energy sources (glucose and fructose) for metastasis, growth, invasion and migration purposes (Port et al. 2012; Santos and Schulze 2012).

Likewise, glycerol is the precursor of triglycerides and phospholipids. It is activated by a phosphorylation reaction and forms glycerol-3-phosphate (G-3-P), which is then involved in the carbohydrate and lipid metabolism. Alternatively, glycerol also functions as a shuttle of electrons from the cytosol to the mitochondria by regenerating NAD^+^ from NADH (Laforenza et al. 2016). In both normal and cancer cell lines, glycerol can be used for gluconeogenesis, although the main metabolite used for that purpose is different; probably glycogen. Nevertheless, there is evidence that in cancer cells, a higher than normal plasma concentration of glycerol (comparable in this case to NADES_4_ treatment) contributes to increased glycerol turnover for gluconeogenesis and lipogenesis (Liu et al. 1995; Lundholm et al. 1982). Judging from these facts, a higher cellular tolerance of these carbohydrates-based eutectics is expected, and this is reflected by the IC_50_ values recorded for NADES_1_, NADES_2_, NADES_3_ and NADES_4_.

In contrast, NADES_5_ which boasts of organic acid as raw material, is the most lethal mixture. Dai et al. (2013) listed NADES_5_ as eutectic used by plants for developmental or metabolic purposes. Although this is valid for certain plants tissues—where malonic acid accounts for as much as 4 % of the dry weight and up to 50 % of the total acid content and may be actively used during nitrogen assisted symbiosis or abiotic stress as a defense chemical; the scenario might be slightly different for mammalian cells (Kim 2002). Indeed, in mammalian systems, malonic acid is known to stall the Krebs cycle by inhibiting succinic dehydrogenase (mitochondria complex II); a crucial enzyme for the citric acid cycle and the electron transport chain (Hosoya and Kawada 1958). Consequently, this paralyzes ATP synthesis. Moreover, malonate is known to disrupt glycogenesis, lipid synthesis and carbon dioxide production during glycolysis (Hosoya et al. 1960). It comes as no surprise that calls have been made for malonate to be used as an anticancer agent. As a matter of fact, Fernandez-Gomez et al. (2005) showed that malonate causes SH-SY5Y neuroblastoma mitochondrial failure by inducing a rapid build-up of ROS, which overwhelms mitochondrial antioxidant capacity, ultimately leading to cellular apoptosis.

This shows that with regards to the HBD, the inclusion of organic acids seem to increase the overall toxicity of NADESs. This is consistent with the other cytotoxic reports on DESs/NADESs (Paiva et al. 2014; Radošević et al. 2015; Zhao et al. 2015).

Zhao et al. (2015) observed that NADESs with organic acids as HBDs had a low pH (<6.5); when the optimal growth range for mammalian cells is 7.0–7.4. This change in environmental conditions is partially responsible for the high toxicity of NADES_5_.

DESs investigations led to a similar observation. For instance, Radošević et al. (2015) observed the formation of intracellular calcium oxalate crystals following [ChCl]-[Oxalic acid] DES treatment of CCO and MCF-7 cell lines. Another perspective on organic acids as HBDs was shown by Paiva et al. (2014). The authors examined NADESs toxicity towards fibroblasts like-cells (L929) and reported that the most lethal solvents also had organic acids as HBDs (i.e. tartaric acid and citric acid). However, it must be noted that the solvents with the highest viability, also had organic acids as ingredients, although the remaining constituent was another HBD (sucrose) and not a salt (ChCl) (Paiva et al. 2014). It might be that the devastating effect of organic acids in NADESs is better countered with the use of biomaterials (e.g. sugars).

The arguments above do not presume to provide a complete understanding of the reasons behind the variation in IC_50_ values; but serve to highlight that safer NADESs can be obtained by using biomaterials of cellular necessity. Of course, the interactions of these mixtures and their aggregation on cellular membranes as well as the neoteric properties of NADESs, remain aspects to be investigated. Meanwhile, physical properties of NADESs can also be used to better appreciate the obtained cytotoxic values.

Table 4 lists the known viscosities values at 30 °C of the understudied NADESs. The values for NADES_1_, NADES_2_, and NADES_5_ were obtained from a recent study by Zhao et al. (2015). The viscosities of NADES_3_ and NADES_4_ were measured independently during this study. Sorting out viscosities in a decreasing order (NADES_3_ > NADES_5_ > NADES_2_ > NADES_1_ > NADES_4_) reveals that they form a series almost similar to the cytotoxicity trend above.

**Table 4:** Viscosities of the understudied NADESs

NADES	Molar ratio	Viscosity (mPa s)	References
NADES_1_	5:2:5	584	Zhao et al. (2015)
NADES_2_	5:2:5	598	Zhao et al. (2015)
NADES_3_	4:1:4	853.3	–
NADES_4_	1:2:1	36	–
NADES_5_	1:1	616	Zhao et al. (2015)

According to Table 4, NADES_5_ and NADES_3_ possess the highest viscosities at 30 °C (respectively 616 and 853.3 mPa s). It is no surprise that they also possess the lowest IC_50_ values on average across all examined cells, as high viscosity is often associated with increased lethality. Despite being less viscous than NADES_3_, NADES_5_ was identified as the most toxic material tested, with an IC_50_ interval (15 ≤ IC_50_ ≥ 35 mM) at least three times lower than NADES_3_’ interval (136 ≤ IC_50_ ≥ 166 mM).

In a separate study, upon testing numerous DESs and NADESs (including similar NADES_1_, NADES_2_, and NADES_5_ used in this study) on several bacteria species (i.e. *Escherichia coli*, *Staphylococcus aureus*, *Salmonella enteritidis*, *Listeria monocytogenes*), Zhao et al. (2015) also identified NADES_5_ as one of the most toxic mixture. In contrast, NADES_1_ and NADES_2_ were found to be non-inhibitory to all studied bacteria. Moreover, the most toxic DESs and NADESs ([ChCl]-[Toluenesulfonic acid], [ChCl]-[Oxalic acid], [ChCl]-[Levulinic acid], [ChCl]-[Malonic acid], [ChCl]-[Malic acid], [ChCl]-[Citric acid], [ChCl]-[Tartaric acid] all included organic acids as HBDs (Zhao et al. 2015). Just as in our study, where, the malonic acid based NADES was the most toxic. This stipulates that despite resulting in high viscosities (compared to water and VOCs), NADESs composed of sugars are relatively less dangerous to biological machinery than those composed of organic acids.

On the other hand, NADES_4_ exhibited the lowest viscosity at 30 °C (36 mPa s) as well as the highest cytotoxicity values. With reference to Table 4, NADES_2_ is slightly more viscous than NADES_1_. Their cytotoxicity values are roughly similar if the SEM is taken into account. Viscosity or micro-viscosity (in cellular terms) is an important property to consider in intracellular activities. Not only does it affect diffusion within biological systems, but it is also involved during processes such as protein–protein interactions, transportation of small solutes and macromolecules, and signal transduction in living cells. The local micro-viscosity in cells ranges from 1 to 400 mPa s (Liu et al. 2014). The highest viscosity values (≥200 mPa s) are usually associated with the microviscosity in the hydrophobic domains of living cells (lipid bilayers of cell surfaces); whereas, values between 1 and 3 mPa s are attributed to the aqueous phases of the cellular cytoplasm (Juneidi et al. 2015; Kuimova et al. 2009). A variation or a disturbance of these homeostatic values leads to the onset of various diseases (atherosclerosis, diabetes) as well as cell death (Deliconstantinos et al. 1995; Nadiv et al. 1994). The understudied NADESs possess viscosities higher than 500 mPa s; with the exception of NADES_4_ (36 mPa s), which happens to be the least toxic mixture tested (with IC_50_ values 1.6–32 times lower than the other NADESs). Hence, it is not difficult to perceive the substantial influence that these highly viscous materials can have on cells. Just like DESs perforate cellular membranes (Hayyan et al. 2015), NADESs can probably enhance cellular membrane permeability. As such, the introduction of such viscous substances in cellular medium can result in a major variation of cytoplasmic microviscosity, and eventually lead to cell death.

Based on the available knowledge of DESs, the high viscosities of NADESs originate from the rigidity of their supramolecular complexes reposing on a strong hydrogen bond network. It entails that the disruption of this network will affect the viscosity of NADESs. In fact, Dai et al. (2015) recently provided evidence of the progressive rupture of this hydrogen bond network upon addition of water. The authors also showed that the supramolecular complexes of NADESs remain intact if the volume of added water is less than 50 %. Pass this threshold, the resulting mixture consists merely of dissociated NADESs ingredients. This is a consequence of the complete rupture of hydrogen bonds stabilizing NADESs. The fact that the entire NADES structure repose on hydrogen bonds means that their progressive breakdown simultaneously induces a change in their physicochemical properties. Accordingly, Dai et al. (2015) reported a decrease in viscosity from 397 to 7.2 mPa s, following the addition of 25 % of water to [ChCl]-[Glucose]-[Water] (in this case NADES_2_).

This argument is further justified by the fact that we have independently recorded a value of 36 mPa s for NADES_4_ ([ChCl]-[Glycerol]-[Water]) viscosity; whereas Zhao et al. (2015) recorded a value of 177 mPa s for the [ChCl]-[Glycerol] DES (both at 30 °C). The effects of water can also be acknowledged through the observation that of all the understudied NADESs, the most toxic and most viscous was the one prepared without the use of water (NADES_5_). Moreover, Dai et al. (2015) stated that the water activity of NADESs increases with increasing water content (or after water addition). Consequently, the polarity of the eutectics after addition of water may mimic that of water itself. This may influence the interactions of these solvents with cell surfaces.

The importance of these interactions must be dully underscored, as DESs have shown that they promote cellular failure through an increase in membrane porosity (Hayyan et al. 2015). In order to have an idea of what unfolds upon NADESs treatment on cell surface, we used a computational methodology using the COSMO-RS software. COSMO-RS is a very useful and fast tool for the prediction of thermophysical and chemical properties of fluid mixtures (Klamt 2005). It is a model that combines an electrostatic theory of locally interacting molecular surface descriptors with statistical thermodynamics. Although mostly used to predict the thermodynamic properties of a mixture without prior experimental data; it can also be applied to life sciences and molecular studies. Examples include the prediction of drug’s partition coefficients and the computation of proteins pKa (Andersson et al. 2013; Buggert et al. 2009). Two major steps are involved in the COSMO-RS prediction process. The first step involves the creation of virtual conductor surroundings for the molecule by using the continuum solvation model. After performing the quantum chemical calculation through the density functional theory, a screening charge density known as sigma (σ) forms on the nearby conductor. The distribution of the screening charge density on the surface of the molecule is then converted into a function of surface composition, known as the σ-profile. The second step applies statistical thermodynamic principles to compute the molecular energy due to the electrostatic misfit, hydrogen bond, and Van der Waals interactions (Klamt and Eckert 2000). COSMO-RS can also be used to study the possible thermodynamic behaviour of an individual component in a mixture and its affinity or interactions with the other components through the σ-profile and σ-potential, respectively. The σ-profile describes the molecule polarity properties. Each peak in the σ-profile plot for a molecule corresponds to its constituent atoms depending on their screening charge densities. The negative partial charges of atoms cause positive screening charge densities, and vice versa.

**Fig. 2:**
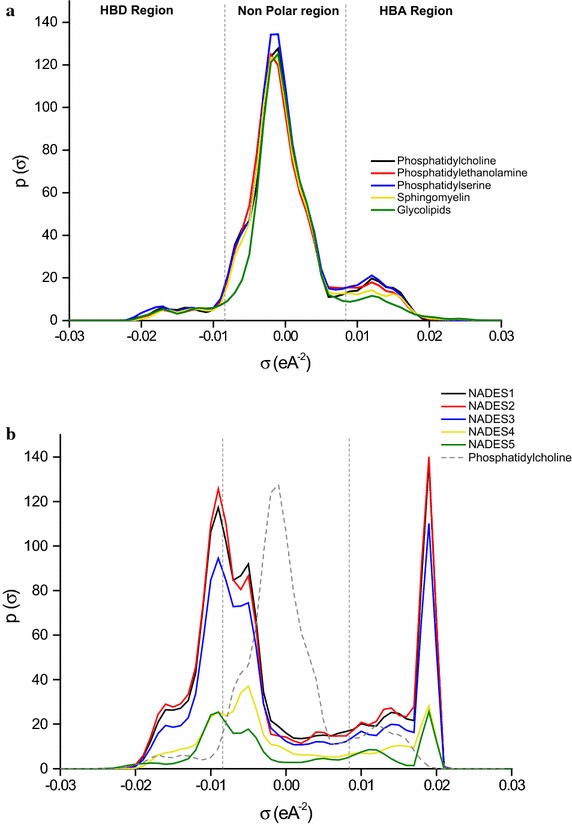
σ-profiles of phospholipids and NADESs. **a** Phospholipids σ-profiles are shown on their own, whereas **b** NADESs σ-profiles with phosphatidylcholine for assessment purposes

Using the elements in Table 2, we modeled each of the listed phospholipids and examined their interactions with the NADESs. The sigma profiles of both phospholipids and NADESs are shown in Fig. 2. For comparison purposes, we only used phosphatidylcholine, as it is the most common membrane phospholipid, and also because, the phospholipids’ σ-profiles and potentials are almost identical.

In the σ-profile, when the screening charge density is lower than −0.0084 eÅ^−2^ or exceeds +0.0084 eÅ^−2^; the molecule is considered sufficiently polar to induce hydrogen bonding.

Figure 2 is divided into three quadrants with corresponding σ values; the HBD region (σ < −0.0084 eÅ^−2^), the nonpolar region (−0.0084 ≤ σ ≤ 0.0084 eÅ^−2^) and the HBA region (σ > 0.0084 eÅ^−2^). Negative values represent positive polarities and vice versa. Hence, the elements in the HBD region of a molecule interact or attract elements in the HBA region (from another molecule) since they are of opposite polarities. Looking at Fig. 2a, the bulks of the peaks reside in the nonpolar region. This is most likely due to the long hydrophobic fatty acid chains making up these phospholipids and included during our modeling. Compared to NADESs, phosphatidylcholine σ-profile (similar to all other phospholipids) breadth is narrower; this is usually indicative of a less polar character (Fig. 2b) (Mulyono et al. 2014).

**Fig. 3:**
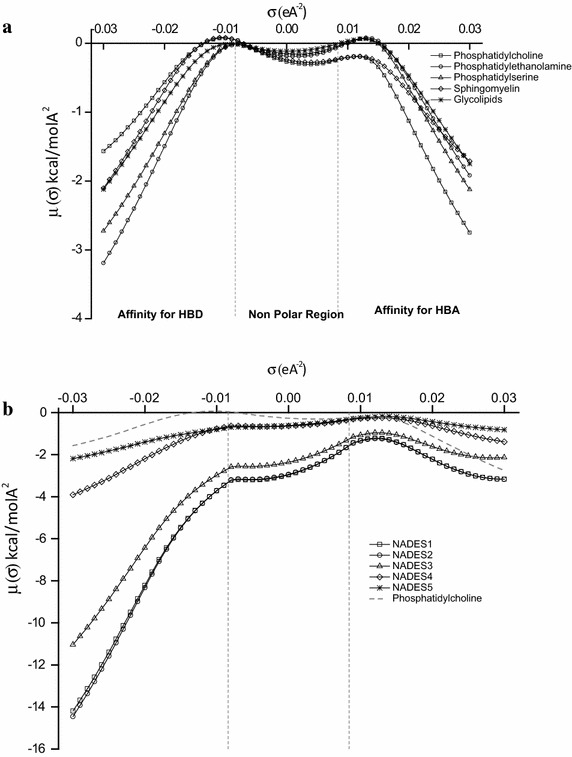
σ-potentials of phospholipids and NADESs. **a** Phospholipids σ-potentials, **b** NADESs σ-profiles with phosphatidylcholine

The peaks located between +0.01 and +0.02 eÅ^−2^ in Fig. 2a, represent the negative charge of the O atoms present in the hydroxyl groups of the phospholipids ingredients (phosphate, glycerol, fatty acid). These O atoms may interact to form hydrogen bonds with the H atoms in NADESs that produce the peaks between −0.02 and −0.01 eÅ^−2^. These peaks belong to the hydroxyl groups of the sugars, polyols, or acids ingredients of NADESs and the H atom of ChCl. Likewise, the broad HBD region of the phospholipids between −0.01 and −0.02 eÅ^−2^ represent H atoms in the glycol groups of glycerol or in the functional group of the acids. These may interact with the elements in the HBA region (which mainly comes from Cl atoms of ChCl at 0.02 eÅ^−2^) in Fig. 2b to form hydrogen bonds;. The importance of these interactions is perhaps best reflected in the σ-potentials of Fig. 3. The σ-potential represents the interaction behavior and affinities between molecules in a system. On the σ-potential plot, a more negative value of µ (σ) indicates higher affinity, and vice versa. Figure 3a shows the σ-potentials of modelled phospholipids. The phospholipids show clear and strong affinities for HBD on the left side, and HBA on the right side, given their high outer negative ranges. Consequently, they will be more attractive for HBDs and HBAs of other molecules. Their nonpolar surfaces possess slightly negative values, which promotes average to low interactions with other molecules nonpolar surfaces. NADESs, in contrast, vary slightly in their σ-potentials. NADES_1_, NADES_2_, and NADES_3_, all possess very strong affinities for HBDs; relatively weaker affinities for HBAs, and low affinities for nonpolar surfaces. When compared to phosphatidylcholine (Fig. 3b), NADES_1_, NADES_2_, and NADES_3_ affinities for HBD, and nonpolar regions is significantly higher; although relatively similar for HBA. It entails that these three NADESs can interact strongly with HBDs, HBAs and nonpolar surfaces of phospholipids than NADES_4_ and NADES_5_. These interactions may correlate with solvent accumulation and aggregation on the cell surface, which ultimately leads to cellular demise through reduced growth. An example of such critical interactions between groups of opposites polarities and affinities was shown by Cornmell et al. (2008). The authors emphasized that the interactions taking place between aqueous quaternary ammonium salts cations (such as cholinium cations) and the negatively charged groups present on cell surfaces may lead to the penetration of the latter in the cytoplasm. The consequences range from the loss of membrane integrity to a subsequent demise of the cell, through an increased permeability of the cell membrane to exogenous species (Cornmell et al. 2008).

NADES_4_ and NADES_5_ affinities are almost similar to phospholipids (phosphatidylcholine). These mild affinities of NADES_4_ and NADES_5_ -for HBDs and HBAs of phospholipids-suggest that perhaps their cytotoxic mechanism is not entirely focused on cellular aggregation, but rather depends on the resulting reactions engendered by the cellular adsorption. That is, Krebs cycle stalling and acidosis by NADES_5_, and cellular poisoning by NADES_4_ at a threshold concentration. It is interesting though to note that the solvents with the overall lesser affinities are both the most toxic and least toxic understudied eutectics.

Of course, this model is not an exact replica of what is found in membranes, especially in terms of ratio and functional groups occurrence. As such, the higher ratio of ChCl, or water and sugars in NADES_1_, NADES_2_, and NADES_3_, and the hypothetical ratio of cell membrane elements, may explain the resulting fluctuating affinities. Phospholipids elements especially, consist of a set ratio of functional groups on the cell surface (carboxyl, phosphate and amino groups). The ratio of these functional groups dictates the entry and the rate of passage of extracellular materials (such as NADESs’ species) in the intracellular medium, as their proportions differ according to cell type. These proportions regulate the diffusion of NADESs, and indirectly affect their effect on the cellular machinery.

The propensity of NADESs/DESs species to permeate through cellular membranes was suggested to obey a principle of colloidal biology, which is based on the Hofmeister phenomenon (Vlachy et al. 2009). An elucidation of the specifics of the principle of affinities between chaotropic and kosmotropic DESs/NADES species and cell surface groups would provide a strong tool for the prediction of the toxicity of these mixtures.

## Conclusion

NADESs show similar physical characteristics to DESs. They exhibit high viscosities, poor conductivities and malleable densities at room temperature. These characteristics are determined by the strong hydrogen networks holding together their supramolecular structures. Loosening this network brings about ideal conditions for the industrial use of these solvents. Changes in temperatures alter this network but so does the inclusion of water as a tertiary component. This study showed that NADESs are generally less toxic than DESs. Moreover, it emphasized the significant role of HBDs with regards to NADESs cytotoxic profiles. The use of biomaterials appears to be an important asset for lowering their cytotoxicity. Organic acids, as in previous reports, should be used with caution as they increase the deleterious attributes of NADESs. The COSMO-RS based computational approach proposed a hypothetical cytotoxic mechanism of NADESs mostly based on cellular aggregation. Although further assessment is needed to draw a comprehensive picture of the cytotoxicity mechanism of these neoteric mixtures; the results obtained in this work are encouraging with regards to their safety.
